# Damage Progress Classification in AlSi10Mg SLM Specimens by Convolutional Neural Network and k-Fold Cross Validation

**DOI:** 10.3390/ma15134428

**Published:** 2022-06-23

**Authors:** Claudia Barile, Caterina Casavola, Giovanni Pappalettera, Vimalathithan Paramsamy Kannan

**Affiliations:** Dipartimento di Meccanica, Matematica e Management, Politecnico di Bari, Via E. Orabona 4, 70125 Bari, Italy; claudia.barile@poliba.it (C.B.); casavola@poliba.it (C.C.); pk.vimalathithan@poliba.it (V.P.K.)

**Keywords:** AlSi10Mg, SLM, NDE, acoustic emission, deep learning, CNN, k-fold cross validation

## Abstract

In this study, the damage evolution stages in testing AlSi10Mg specimens manufactured using Selective Laser Melting (SLM) process are identified using Acoustic Emission (AE) technique and Convolutional Neural Network (CNN). AE signals generated during the testing of AlSi10Mg specimens are recorded and analysed to identify their time-frequency features in three different damage evolution stages: elastic stage, plastic stage, and fracture stage. Continuous Wavelet Transform (CWT) spectrograms are used for the processing of the AE signals. The AE signals from each of these stages are then used for training a CNN based on SqueezeNet. Moreover, k-fold cross validation is implemented while training the modified SqueezeNet to improve the classification efficiency of the network. The trained network shows promising results in classifying the AE signals from different damage evolution stages.

## 1. Introduction

The evolution of Additive Manufacturing (AM) technique is a narrative of its own. Its application began with complicated geometries and functional prototypes. In the last decade, it has evolved into one of the key manufacturing systems for many industrial and aeronautic components. The functionality of the AM components has made them enter into the maritime applications [1,2,3,4]. Consequently, an intensive scrutinizing is required for validating the safety of the AM components. 

Selective Laser Melting (SLM) is a predominantly used AM technique, known for its good quality, short lead times, limited restriction and high resolution for complex shapes and structures [5]. A very fine-grained structure can be achieved through the SLM process, which results in the improved mechanical properties of the fabricated components. A wide range of materials including Ni, Al, Ti alloys can be manufactured using the SLM process. The components manufactured from the SLM process have relatively improved mechanical properties, corrosion resistance and fatigue life compared to the traditionally manufactured components. The fine-grained structure of SLM manufactured components is supposed to give better isotropic properties, but there has been a long-standing debate on this. SLM components generally have isotropic strength; however, they have a higher anisotropy in the elongation at break [6,7]. The isotropic behaviour of the SLM components is generally related to their building direction. A homogenous microstructure provides high mechanical strength to the components and can accommodate strain which can reduce cracking. However, due to the high cooling rates in the SLM process, which is in the range of 10^6^–10^8^ °C/s, achieving equiaxed grain is a challenge [5,8,9,10,11]. Thus, it is essential to study the behaviour of SLM components built in different direction. 

In this study, the mechanical characteristics of the SLM components built from AlSi10Mg alloy at different orientations are studied. Acoustic Emission (AE) technique, a Non-Destructive Evaluation (NDE) technique, is used for studying the damage behaviour of the SLM components. The AE technique is based on the acquisition of elastic waves generated due to the rapid release of stored elastic energy [12]. Microscopic displacements within a solid material due to microcracking, crack nucleation or crack growth generate elastic waves. Each of these microscopic damages within a material generates acoustic waves that differ in their time-frequency characteristics. Analysing these acoustic waves to identify the damage source and predicting the failure is the basis of the AE technique [13,14,15]. This is one of the few available passive techniques for investigating the damage characteristics of a material throughout its loading history. 

Time-frequency analysis of stress waves generated from Fibre Reinforced Polymer (FRP) composites is relatively common [16]. However, it is seldom used for characterizing the damage modes in metallic components. In the authors’ previous studies, time-frequency analysis of acoustic waves has been successfully used in the identification of damage sources in metallic components [17].

In the recent years, Deep Learning has been used contemporarily with the AE technique for damage characterization [18]. Convolutional Neural Networks (CNN) have been used for identifying damage modes in SiC composites, CORTEN steel and civil structures [19,20,21,22,23,24,25], while Artificial Neural Networks (ANN) have been used for corrosion monitoring of steel. While both these networks can relate a large number of parameters and building a model for classification and prediction in real-time, CNN is preferred generally for image-based analysis. While there is an argument that ANN such as Long Short-Term Memory (LSTM) have advantages over the feed-forward neural networks such as CNN, many literatures have used CNN for image-based analysis [26,27]. LSTM is suitable for handling temporal or sequential data, while CNN are used predominantly for image-based analysis. Apart from these, there are several other image-based and wavelet-based neural networks, which have been used successfully in the past [28].

Acoustic signals generated from different damage modes have their unique time-frequency signatures. This can be observed and analysed in their time-frequency spectrum using wavelet spectrograms. The spectrograms are images containing the spectral details of the analysed waveforms. In that context, they can be used as inputs to train neural networks. Previously, this technique has been used successfully in identifying the damage modes in FRP composites, SiC composites, and civil structures [21,23,29]. The aim of this research is to use the image-analysing capability of CNN for classifying the acoustic emission signals from different damage modes. This is possible by training a CNN with time-frequency spectrograms of AE signals generated from various damage sources of a material/structure. 

The objective of this research is to investigate the mechanical properties of SLM manufactured AlSi10Mg components in different orientations. In addition to that, the AE technique is used for identifying the damage sources and a Deep Learning neural network is modelled and trained for real-time identification of damage stages.

## 2. Materials and Methods

### 2.1. Materials

The AlSi10Mg alloy used in this study for manufacturing has a density of 2.68 g/cm^3^ and a melting range of 570 °C to 590 °C. The chemical composition of the feed material is presented in Table 1.

The SLM manufacturing system is RenAM 500 M (Renishaw S.p.A., Torino, Italy), in which an Nd:YAG laser (wavelength 1.064 μm) is used for melting the feed material. Dog-bone shaped tensile specimens are prepared on the heated bed inside the SLM chamber. The recoater moved along the *Y* axis to coat the powder, then the laser moved along the *X* axis to melt the powder. The laser source of power 400 W created a single-track energy density of 20 J/mm^2^ melted the coated powder. The laser beam of spot diameter 200 μm and the speed of the laser movement along the *X* axis is 100 mm/s. The powder is melted for form a uniform layer of 20 μm thickness, before the recoater coats the powder again. The process is repeated until the complete specimen is built. 

**Figure 1:**
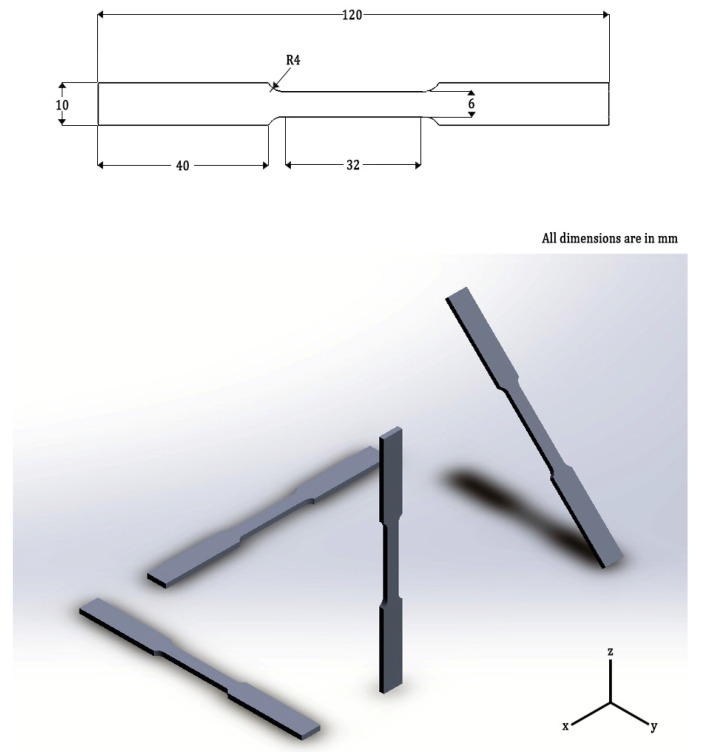
Dog-bone shaped specimens built in four different configurations in SLM building platform and their dimensions.

Dog-bone shaped specimens as per ASTM E8M configurations are built along four different orientations, which are displayed in Figure 1. The different orientations are achieved by moving the powder bed, while keeping the laser axis the same throughout the process. At the end of the process, the specimens are kept inside the environmental chamber of the SLM system at 300 °C for 2 h and then they are air-cooled.

### 2.2. Test Methods 

For testing the mechanical properties of the specimens, they are mounted on an INSTRON servo-hydraulic testing machine (Norfolk County, MA, USA), with a maximum loading capacity of 10 kN. The tensile tests are carried out at a speed of 1 mm/min as per ASTM E8M standard.

For recording the acoustic waves/stress waves generated due the damage evolution during the tensile test, a piezoelectric sensor is coupled to the surface of the specimen. A uniform thin layer of silicone grease is applied between the surfaces of the sensor and the specimen. The PICO sensor (Physical Acoustics Corporation, Princeton JCT, USA) is a wideband sensor with the maximum sensitivity in the acquisition range of 200 kHz to 750 kHz and it has a resonant frequency of 250 kHz. The signals recorded by the sensors are amplified by 40 dB using a preamplifier and filtered through 1 kHz/1 MHz low/high-band pass filters. The signals are recorded at a sampling rate of 1 MHz.

### 2.3. Time-Frequency Analysis of AE Signals

The acoustic signals recorded over the entire loading history of the tensile test of AlSi10Mg specimens are analysed in their time-frequency domain to understand their damage sources. The time-frequency characteristics of the acoustic signals are analysed using the signal processing technique, Continuous Wavelet Transform (CWT) [30,31]. First, CWT is used for identifying the damage modes; second, the spectrograms are used for training and testing the image-based classification neural network. Researchers commonly use waveforms in their time-series representations or spectrograms of CWT, Short-time Fourier Transform (STFT) or Mel Spectrograms [21,22,23]. The independence of selecting a user-defined wavelet for decomposing the signal makes CWT a formidable signal processing tool. It has been used successfully by several researchers for damage characterization of FRP composites using AE technique. 

In CWT, the original signal f(x) is decomposed into wavelet coefficients using a mother wavelet. The wavelet coefficients give information about the time-frequency localization of the spectral components of the original signal [31]. CWT can be explained by Equation (1).

(1) $$CWT_{f}\left(a,b\right)=\int_{-\infty }^{\infty }f\left(x\right)\bar{\zeta_{a,b}\left(x\right)dx},\;a>0,$$

a in Equation (1) is the scaling factor with which the mother wavelet $\zeta_{a,b}\left(x\right)$ is dilated or compressed and b is the translation factor, which gives the wavelet components in different time domains. The mother wavelet used in this study is an analytical Morlet Wavelet, which can be defined by Equation (2) [32].

(2) ζ(x)=exp(−x22)cos(5x),

Literatures are enriched with the basic principle and implementation of CWT technique for time-frequency analysis [30,31]. In this research, this process is carried out in MATLAB^®^ (2020b). 

### 2.4. Convolutional Neural Network

Generally, CNN consists of an input layer, a classification layer and a set of hidden layers. One of the most important hidden layers is a convolutional layer. Convolutional layer features a set of weighted filters, which extract the feature map from its input. The output of the convolutional layer is generally followed by a pooling layer and an activation function. The pooling layer extracts the most representative features of the convolutional output by padding and striding operations. Max pooling and average pooling are the commonly used pooling operations in a CNN. The most used activation functions are sigmoid, tanh and ReLu activations. Stochastic Gradient Descent (SGD) is typically used for training the models. ReLu activation function is preferred for SGD training algorithms because of its non-saturation of gradients and the efficient convergence in SGD [33]. 

Typically, the number of hidden layers is based on the depth of the features to be extracted from the input image. However, if more layers are used, overfitting may occur, and the classification accuracy is ultimately affected. To avoid the overfitting of the results, a dropout layer can be used. 

Branching and exploring multiple paths of the hidden layers are explored by many researchers to obtain the highest classification accuracy. This also can extract different levels of abstraction and enables the network to learn the information in the early stages, which flows the information more easily into the classification layer. 

More deep layers and branching makes the computation time longer and requires large computation power. To overcome this problem, Iandola et al. introduced a fire module [34]. This contains a squeeze convolutional layer, which has a 1 × 1 filter, whose output is fed to an expanding convolutional layer, which is a mix of two convolutional layers having 1 × 1 filter and 3 × 3 filter, respectively. The idea is to use 1 × 1 filters for most of the convolutional layer and create a large activation data pool by delaying the downsampling of data. Most commonly the downsampling is done by setting the stride value in the CNN architecture to be greater than 1. In SqueezeNet, however, the stride value is kept to 1. The pooling layer always follows the convolutional and activation layer. More details about this SqueezeNet architecture can be found in the source paper. 

In this research, a similar CNN based on the SqueezeNet is used for classifying the waveforms with high accuracy. To avoid the overfitting of data, some of the deeper convolutional layers are removed from the original network, but the sizes of filters are increased in the deeper convolutional layers. In the original network, there were 68 layers and 75 connections. It had 8 fire modules for extracting the deeper features. In the modified network, there are 39 layers with 42 connections and 4 fire modules. However, large filter sizes are used in the deeper convolutional layers, similar to the original SqueezeNet. Subsequently, a moderately large activation pool is obtained. The dropout layer is used for avoiding the overfitting of data. 

**Figure 2:**
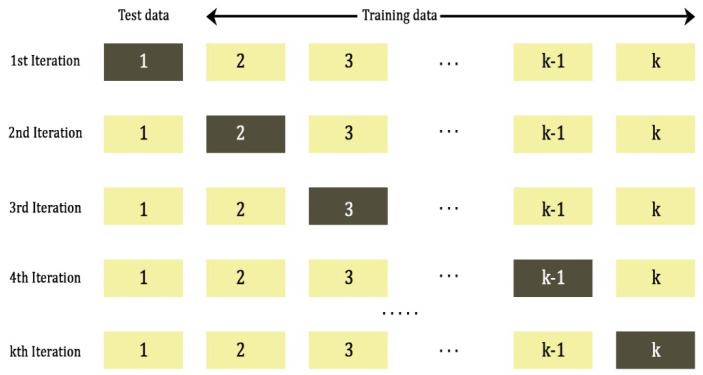
Schematic of k-fold cross validation.

In this study, to improve the training efficiency of the SqueezeNet, k-fold cross validation is implemented. k-fold partitions the input data to the network into k number of subsets. For each iteration of training the network, (k−1) number of subsets are used as a training data and the remaining subset is used as a test data [35,36]. This reduces the bias as most of data are used for training the network for k iterations. Besides, the network weights of the convolutional layers are updated constantly for each iteration, thereby improving the efficiency of training. Typical k-fold configuration is presented in Figure 2. In this study, the input data is split into k=5 subsets and the network is trained for 5 iterations.

**Figure 3:**
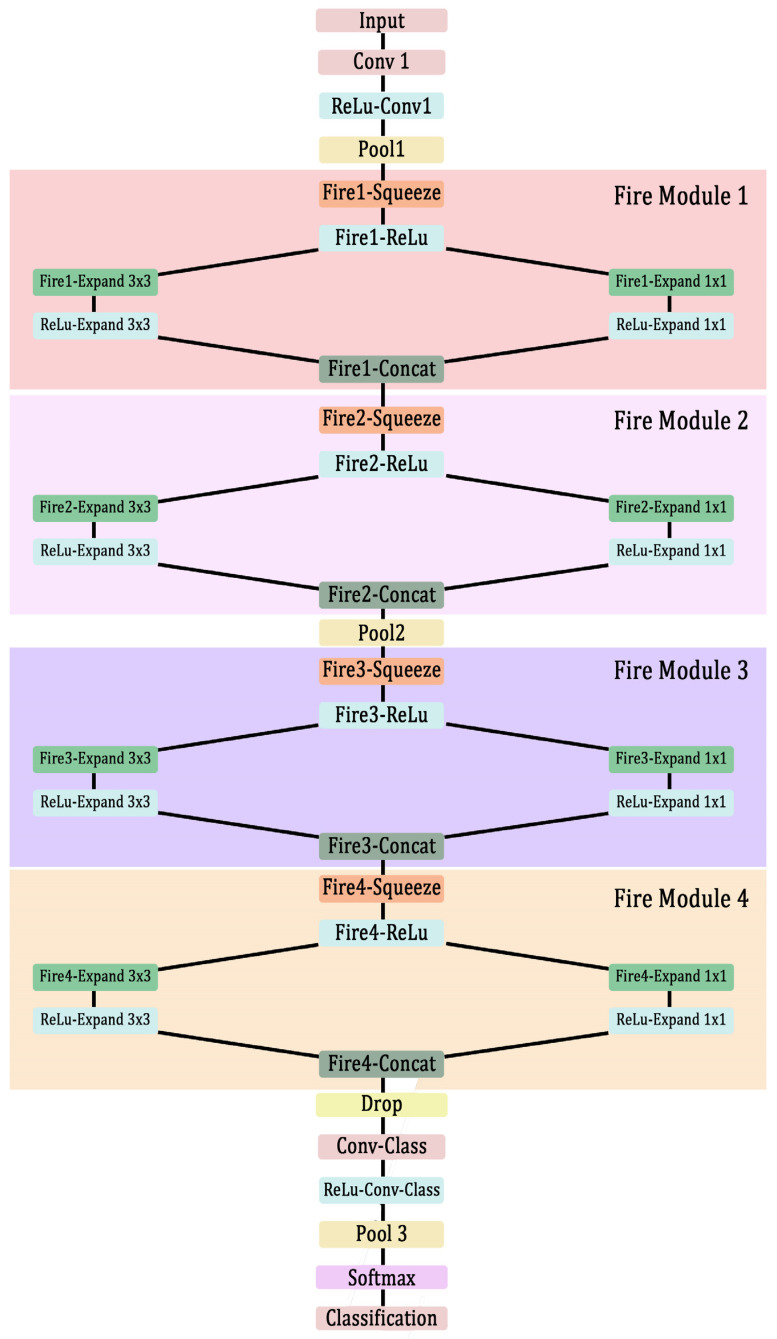
Architecture of the modified SqueezeNet (data flowing in one direction—from input layer to classification layer).

The architectural details of the CNN built for this study are presented in Figure 3. The configuration of a typical fire module consisting of squeezing and expansion of the convolutional output is explained in Table 2.

In Table 2, the *n* represents the number of the fire module layer, which can be seen in Figure 3. X is the number of filters in each layer. More details about the number of filters in each layer and other features such as filter size and stride are presented in Table 3.

The data used for training the CNN and for evaluating its efficiency are presented in the Results and Discussions section.

## 3. Results and Discussions

### 3.1. Tensile Test Results

Four different configurations of SLM specimens based on different build orientations (named as Tx, Ty, Tz and T45) are tested. Six specimens from each group are tested. The mechanical properties, ultimate tensile strength, yield strength, Young’s modulus, and elongation at break of the specimens are reported in Table 4. These properties are calculated according to the ASTM E8 standard.

For all the four groups of specimens, the ultimate tensile strength, yield strength and Young’s modulus are quite similar. However, the elongation at break of specimen groups Tz and T45 specimens are quite low in comparison with Tx and Ty. It has been reported by several researchers that the changing in build orientation can result in the microstructural heterogeneity of the SLM components [10]. Dong et al. studied the thermal transfer mechanisms of AlSi10Mg specimens and derived the microstructural variations due to the build orientations of the components [8,9,11]. This probably could be the reason for the large variation in elongation at break between the specimens. 

More details about the mechanical results and their dependence on the build orientations can be found in the authors’ previous research works [17,37,38]. In this work, the different damage evolution stages in these specimens are analysed using the AE technique and deep neural network model.

**Figure 4:**
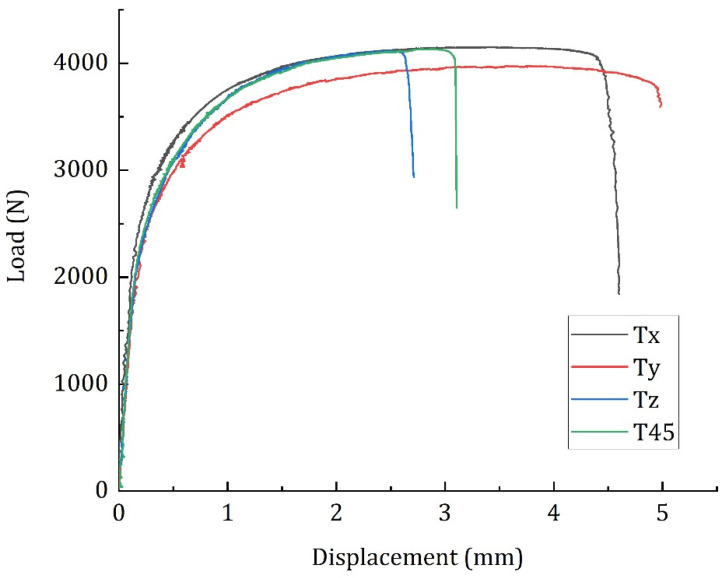
Load-displacement curves of the AlSi10Mg specimens built in four different orientations.

Representative load-displacement curves of the four different specimen groups are presented in Figure 4. All the four specimens apparently show a similar load response during the tensile tests. Until the yield point, these specimens show a linear elastic behaviour, which is followed by a yield phase. As observed in Table 4, the duration of the yielding phase varies between the specimens. Nonetheless, the duration between the commencement of fracture and the final failure is quite similar. It can be assumed that these specimens have three damage evolution stages: elastic stage, plastic stage, and fracture stage.

**Figure 5:**
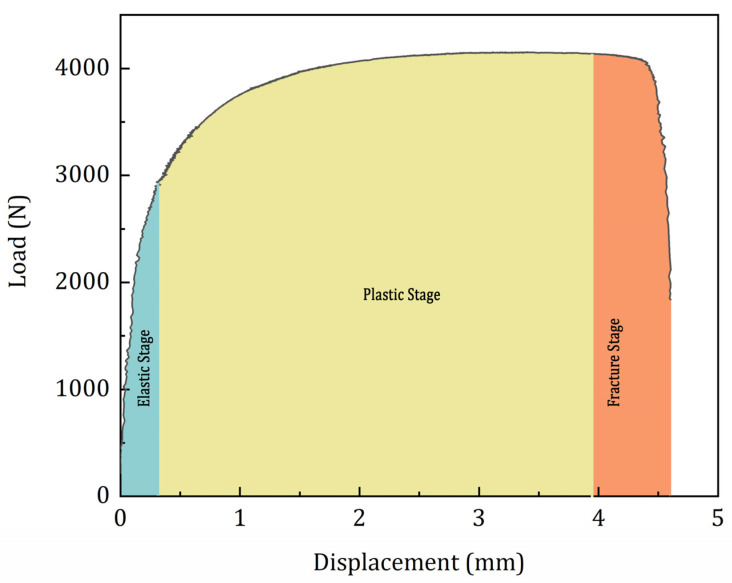
Damage stages considered for the AE analysis.

First, the yield point is selected from the stress–strain curve of the tensile test data as per the instructions in ASTM E8 standard. The region until the yield point is considered as the elastic stage, where there is a linear elastic stress response by the specimen to the applied load. Second, the plastic stage is selected from the yield point until the region where the slope of the load response starts to decrease. Finally, the region beyond the plastic stage until the final fracture is considered as the fracture region. A schematic representation of the different damage evolution stages is presented in Figure 5. AE signals recorded from each of these stages are collected and are analysed in their time-frequency domain.

### 3.2. Time-Frequency Characteristics of AE Signals from Different Damage Modes

**Figure 6:**
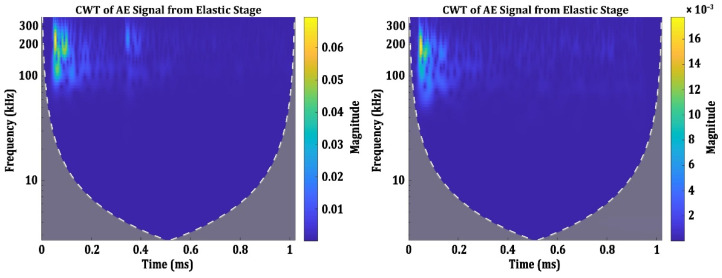
CWT Spectrograms of the AE signals from the elastic stage.

AE signals from the elastic stage of all four groups of specimens Tx, Ty, Tz and T45 are collected and analysed in their time-frequency domain using CWT. Interestingly, all the AE signals from the elastic stage can be grouped into two categories based on their time-frequency characteristics. The CWT spectrograms of these two categories of signals are presented in Figure 6. 

It can be observed that in both the categories, most part of the spectral components are present in the same frequency (between 200 kHz and 250 kHz) [39] and are localized in a similar time domain. The only difference between these two categories is their magnitude. The maximum magnitude of AE signals in the first category is between 0.06 and 0.08, while the second category is generally between 0.01 to 0.02. Nonetheless, these are the typical characteristics of the AE signals from the elastic stage. There are very few literatures available to compare the frequency components and the magnitudes of AE signals generated during the linear elastic response of metallic components [40]. However, it has been reported by some researchers that during the elastic stage, the AE events are generated due to the dislocation motions, and they have frequency components between 200 kHz and 250 kHz. The presence of similar AE signals in all the four specimen groups show that the signals presented in Figure 6 are the general characteristics of the AE signals generated during the elastic stage.

**Figure 7:**
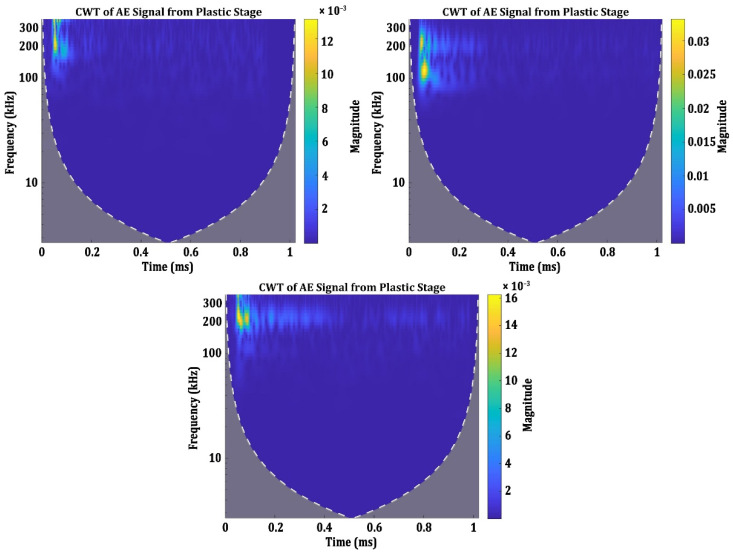
CWT Spectrograms of the AE signals from the plastic stage.

During the plastic stage, however, AE signals are generated due to various damage evolutions in the specimens. Grain boundary movements, local yielding around the inclusions, local plastic deformation around the pores and the plastic deformation are some of the sources of AE signals during the plastic deformation stage [39,40,41,42,43]. While analysing the AE signals in the plastic stage of all four groups of specimens, three different categories of AE signals are observed. These signals can be categorized based on their time-frequency characteristics. The CWT spectrograms of the signals from the plastic stage are presented in Figure 7.

The three categories of AE signals in the plastic stage have obvious differences in their frequency characteristics. The first category of the signals in the plastic stage has two frequency components, one localized around 100 kHz and the other around 200 kHz. The second category of AE signals has its spectral energy centred in the frequency band above 300 kHz and the signals are localized in a very narrow time domain compared to the other categories. The third category has its spectral energy centred between 200 kHz and 250 kHz. However, these signals have lots of reverberations, which can be seen by the similar spectral components with lesser magnitude appearing up to the signal length of 0.4 ms. In the literature, some of these signal characteristics are observed in the plastic stage of loading. Abkari and Ahmed have observed AE signals with frequencies above 300 kHz in the plastic region [42]. Nonetheless, it can be said that the AE signals generated during the plastic stage have three specific characteristics. 

**Figure 8:**
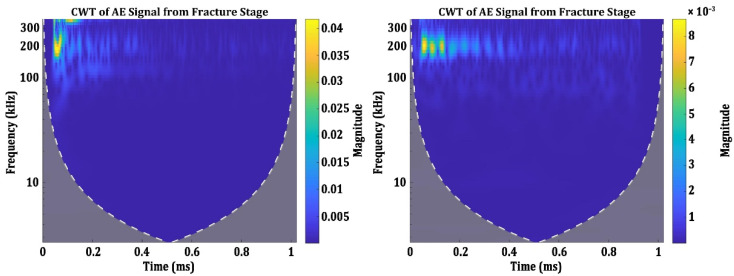
CWT Spectrograms of the AE signals from the fracture stage.

AE signals from the fracture stage show two distinct time-frequency characteristics. The CWT spectrograms of these signals are presented in Figure 8. It can be observed that the CWT spectrograms of the signals in Figure 8 have more reverberations than the signals shown in Figure 6 and Figure 7. This possibly could be due to the overlapping of several signals generated in short intervals in the fracture stage. The final fracture occurs in a very short duration under loading, and this possibly could have generated signals with lots of reverberations. The first category of these signals has its spectral energy distributed in two frequency bands: one at 200 kHz and another above 300 kHz. The second category of the signals have lots of reverberations, but the spectral component is localized at 200 kHz. The reverberations extend up to the length of 0.4 ms of the signal.

### 3.3. Convolutioanl Neural Network Training and Test Results

In the previous section, AE signals from all the specimens are recorded and analysed in their time-frequency domain. Based on their time-frequency characteristics, two categories of signals in the elastic stage, three categories in the plastic stage and two categories of signals in the fracture stage are observed. AE signals having the similar time-frequency characteristics as those displayed in Figure 6, Figure 7 and Figure 8 are used for training the CNN. The objective is to train the CNN to classify these groups of signals automatically and thereby classifying the damage evolution stages of the AlSi10Mg specimens under loading. 

The CNN can be trained more efficiently if the number of training data is high. The number of AE signals recorded during the tensile tests of four groups of AlSi10Mg specimens is less than 5000. When these signals are classified into three damage evolution stages, the plastic stage contains around 3000 signals, while the elastic stage and fracture stage has 1000 signals each. This is not sufficient to train the CNN. Therefore, these signals are augmented by adding random noises. In all, 15,000 signals for each damage evolution stage, a total of 45,000 signals, are generated. A random selection of 30,000 signals is used for training and the remaining 15,000 signals are used for validating its classification efficiency. 

As indicated in Section 2.3, the CWT spectrograms of these signals are used for training and testing the CNN. The CNN is trained for a maximum of 10 epochs with a minibatch size of 100. 

**Figure 9:**
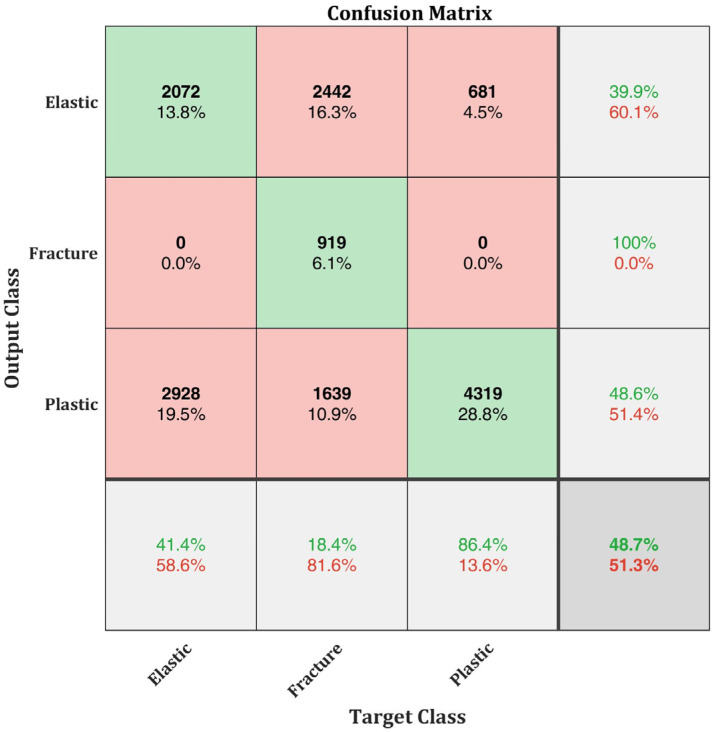
Confusion matrix of the original SqueezeNet without k-fold cross validation.

During the initial analysis, the default SqueezeNet from the MATLAB Deep Network Designer toolbox was used. The training efficiency reached 100% at the end of 10 epochs. The total time for training the original SqueezeNet is 54 min. Although the training efficiency reached 100%, the classification efficiency was merely 48.7% (Figure 9). This is due to the overfitting of data. The original SqueezeNet is more efficient in extracting deeper features in complex images of objects or beings. The input data used in this study are spectrograms. Extracting deeper features in the spectrograms often results in overfitting of data. Therefore, a modified SqueezeNet, as explained in Section 2.4, is trained in this study.

**Figure 10:**
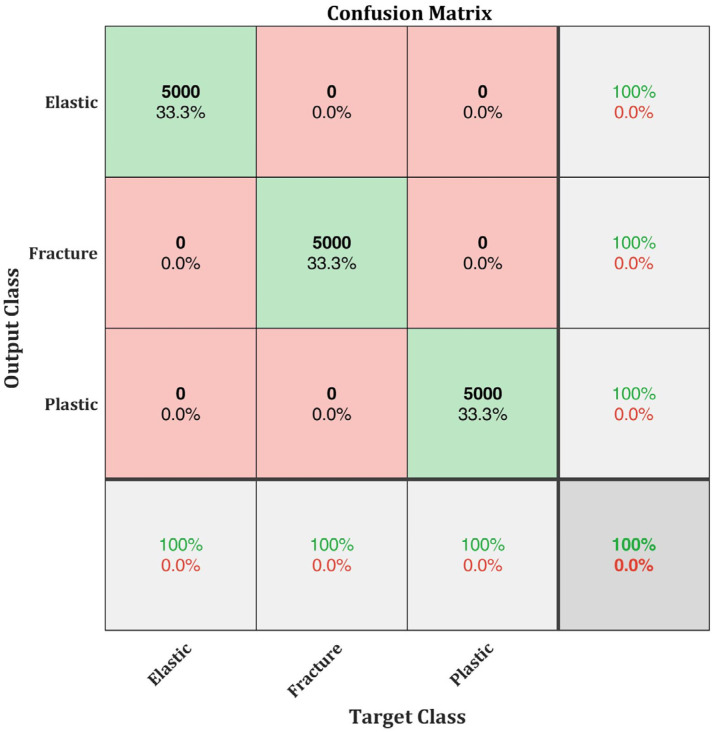
Confusion matrix of the modified SqueezeNet after implementing k-fold cross validation.

After implementing k-fold cross validation into the training module, the classification efficiency of the modified SqueezeNet increased to 100%. The data is partitioned into k = 5 and trained for 5 iterations. Since the training is continued for 5 iterations to improve the classification efficiency, the total time for training this network is approximately 5 times the time taken for training the original SqueezeNet. Nonetheless, considering the increase in classification efficiency from 48.7% to 100%, the total time consumption of 4 h and 12 min is within the acceptable limit. Thus, the modified SqueezeNet with k-fold implementation is considered a relative success. The confusion matrix of the modified SqueezeNet with k-fold implementation is presented in Figure 10. 

The results show that the modified SqueezeNet can explicitly classify the AE signals from the three different damage evolution stages when the k-fold cross validation is implemented. Using this neural network model, the AE signals generated from different damage evolution stages can be identified.

## 4. Conclusions

A modified SqueezeNet neural network with k-fold cross validation is proposed in this research work for identification and classification of AE signals generated from different damage evolution stages of AlSi10Mg test specimens. Four different configurations of AlSi10Mg specimens prepared using SLM process are tested. AE signals generated during the test are recorded and analysed. First, the damage evolution of the specimens is classified into three stages: elastic stage, plastic stage, and fracture stage. AE signals from each stage are extracted and analysed in their time-frequency domain using CWT. AE signals from each of the damage stage showed differences in their time-frequency characteristics, which are used for training the modified SqueezeNet neural network built for this study. The classification efficiency of 48.7% is obtained for the original SqueezeNet without the k-fold implementation and it increased to 100% when k-fold cross validation is implemented for training. The proposed network can efficiently classify the AE signal generated from different damage evolution stages of AlSi10Mg specimens.

## Figures and Tables

**Table 1:** Chemical composition of the feed material AlSi10Mg alloy.

Element	Al	Si	Mg	Fe	N	O	Ti	Zn	Mn	Ni	Cu	Pb	Sn
**Mass (%)**	Bal *	11	0.45	<0.25	<0.2	<0.2	<0.15	<0.1	<0.1	<0.05	<0.05	<0.02	<0.02

* Balance percentage.

**Table 2:** Typical fire module consisting of squeeze and expand convolutional layers.

Fire Module *n*	Layer Name	Layer Description
Squeeze	Fire*n*-Squeeze	Number of Filters: X Filter Size: 1 × 1 Stride: 2 × 2 ReLU activation
Expand	Fire*n*-Expand 1 × 1	Number of Filters: X Filter Size: 1 × 1 Stride: 2 × 2 ReLU activation
	Fire*n*-Expand 3 × 3	Number of Filters: X Filter Size: 1 × 1 Stride: 2 × 2 ReLU activation
Concatenation	Fire*n*-Concat	

**Table 3:** Layer details and descriptions of the modified SqueezeNet.

Fire Module *n*	Layer Name	Layer Description
Input Layer	Input	32 × 32 × 3 Spectrograms
Convolutional Layer	Conv1	Number of Filters: 32 Filter Size: 3 × 3 Stride: 2 × 2 ReLU activation
Max Pooling Layer	Pool1	Pool Size 3 × 3 Stride: 2 × 2
Fire Module	Fire Module 1	Number of Filters in Squeeze: 16 Number of Filters in Expand: 32
Fire Module	Fire Module 2	Number of Filters in Squeeze: 32 Number of Filters in Expand: 64
Max Pooling Layer	Pool2	Filter Size: 3 × 3 Padding: 0,0,0,0 Stride: 2 × 2
Fire Module	Fire Module 3	Number of Filters in Squeeze: 64 Number of Filters in Expand: 128
Fire Module	Fire Module 4	Number of Filters in Squeeze: 128 Number of Filters in Expand: 256
Dropout Layer	Drop	Probability: 0.5
Convolutional Layer	Conv-Class	Number of Filters: 6 Filter Size: 1 × 1 Stride: 2 × 2 ReLU activation
Global Average Pooling Layer	Pool3	-
Softmax Layer	-	-
Classification Layer	-	-

**Table 4:** Tensile test results of AlSi10Mg specimens built in different orientations.

Specimen Name	Ultimate Tensile Strength	Yield Strength	Young’s Modulus	Elongation at Break
	MPa	MPa	GPa	%
Tx	217.2 ± 2.4	137.0 ± 1.5	70.7 ± 3.8	14.2 ± 0.5
Ty	213.6 ± 4.2	132.4 ± 2.4	65.2 ± 1.9	11.2 ± 4.9
Tz	214.4 ± 2.5	126.7 ± 2.8	65.8 ± 1.1	8.9 ± 1.1
T45	218.7 ± 2.4	132.0 ± 2.5	67.4 ± 3.2	7.4 ± 18

## Data Availability

Not applicable.
